# Clinical Approaches of Whole-Body Vibration Exercises in Individuals with Stroke: A Narrative Revision

**DOI:** 10.1155/2018/8180901

**Published:** 2018-09-24

**Authors:** Borja Sañudo, Redha Taiar, Trentham Furness, Mario Bernardo-Filho

**Affiliations:** ^1^Departamento de Educación Física y Deporte, Universidad de Sevilla, Sevilla, Spain; ^2^GRESPI, Research Group in Engineering Sciences, University of Reims Champagne-Ardenne, France; ^3^Australian Catholic University, School of Nursing, Midwifery and Paramedicine, Victoria, Australia; ^4^Laboratório de Vibrações Mecânicas e Práticas Integrativas, Departamento de Biofísica e Biometria, Universidade do Estado do Rio de Janeiro, Rio de Janeiro, RJ, Brazil

## Abstract

Stroke is associated with long-term disability and patients experience numerous physical impairments including muscle weakness, particularly in the paretic limbs, balance, and functional mobility. During acute stroke rehabilitation, when individuals are less likely to be functionally independent and rely on rehabilitative care, the efficacy of low skill interventions that can reduce sedentary behaviour should be established. As such, this narrative revision focused on the use of empirical studies of whole-body vibration exercise (WBVE) on different health outcomes in stroke patients. The effects of WBVE on neuromuscular performance (muscular strength and power), mobility, spasticity, and cardiovascular responses have been highlighted. Although some positive results were reported we can conclude that there is no solid evidence confirming the beneficial effects of WBVE among people with stroke compared with either other types of physical activities or sham WBVE. Therefore, further research should be performed in this area, testing the feasibility and efficacy of using WBVE in a more homogeneous sample of stroke patients or comparing different WBVE parameters.

## 1. Introduction

The relevance of clinical approaches to promote and to maintain health, wellbeing, and quality of life in the context of cardiovascular diseases has been highlighted [1]. Stroke (cerebrovascular accident) is the second leading cause of death and the third leading cause of disability [2]. In this framework stroke was associated, in general, with long-term disability [3]. These patients may experience numerous physical impairments including muscle weakness, particularly in the paretic limbs, balance, and functional mobility [4, 5]. The prevalence of spasticity is also high in this population which can negatively affect gait and cause pain [6] which is strongly associated with limitations in performing activities of daily living [7]. Therefore, stroke has a considerable socioeconomic impact worldwide [8]. Therefore, strategies centred in the prevention and management of this condition are of interest for health care decision makers.

Stroke predisposes an individual to a sedentary lifestyle (especially during acute rehabilitation, when individuals are less likely to be functionally independent and rely on rehabilitative care) that leads to decreases in the cardiorespiratory capacity compared with physically inactive nonclinical individuals [9]. The consequences of this condition persist over time and thus, chronic individuals (with more than 6 months poststroke) have the greatest demand for assistance in their management. Evidence suggests that these physical impairments are, at least in part, reversible with appropriate exercise interventions designed to enhance functional independence in long-term survivors with stroke [10]. Potentially less invasive interventions that could include reducing sedentary behaviour are an important addition to rehabilitation [11, 12] and thus, to face these limitations, numerous forms of training have been suggested, among which we find WBVE as an alternative to other forms of training. As such, this narrative revision focused on the use of empirical studies of whole-body vibration exercise (WBVE) on different health outcomes in stroke patients.

### 1.1. Whole-Body Vibration Exercise

As a relatively simple and low skill demand mode of physical activity, WBVE has primarily been used in athletic [13, 14] and aged [15, 16] cohorts to improve outcomes such as muscular strength and power [17, 18] and reduce sedentary time [13]. Moreover, as a mode of physical activity, WBVE has been identified as a feasible intervention with little to no adverse events [19, 20]. WBVE has progressively been applied with beneficial outcomes to various clinical populations including stroke [7, 21], obesity [22], rheumatoid arthritis [23], diabetes [24], spinal cord injury [25], chronic kidney disease [26], fibromyalgia [27, 28], multiple sclerosis [29], cerebral palsy [30, 31], Duchenne muscular dystrophy [32], osteogenesis imperfecta [33], osteoarthritis [34], and chronic obstructive pulmonary disease [35]. The effects of the WBVE may be due to neuromuscular responses to the interaction of the mechanical vibrations with the body and the tonic vibration response [36–39]. Among clinical populations, various beneficial effects have been associated with WBVE, such as enhancement of the muscular strength and power, improvement of flexibility, gait speed, blood flow, and balance, and diminishing pain and risk of falls. Consequently, the associated improvement in the quality of life in various domains has been reported in various clinical populations [36–40].

Despite these positive effects, the appropriate application of WBVE to clinical populations requires further investigation. There is a lack of synthesis about the safe and effective prescription of WBVE as a mode of physical activity added to rehabilitation. There is a need for clinicians to be provided with clear recommendations about the biomechanical parameters related to the mechanical vibration on the oscillatory/vibratory platform, i.e., frequency (Hz), amplitude (mm), peak-to-peak displacement (mm), and gravitational load (*g*) that will be tolerated by suboptimal health populations during rehabilitation and practice. Moreover, clinicians need clearer recommendations about the duration of exposure to mechanical vibration, rest intervals, type and length of intervention, and body posture on the platform during WBVE [36–39, 41].

Mechanical vibrations are postulated to stimulate the muscle spindles and the alpha motor neurons initiating muscle contractions similar to that of resistance training. Thus it is somewhat understandable that, in the past decade, WBVE has been widely used among people with stroke [10, 42–60]. However, inconsistent results have been observed. Individuals living with chronic stroke are the clinical subgroup that has generated the greatest interest in applications of WBVE. Therefore, the aim of this narrative review was to present the main findings related to the use of WBVE in chronic stroke individuals.

## 2. Effects of Whole-Body Vibration Exercise on Different Health Outcomes in Chronic Stroke Patients

### 2.1. Effects of WBVE on Neuromotor Performance Poststroke

Eight randomized controlled studies have examined the efficacy of WBVE on different aspects of neuromotor function poststroke, such as leg muscle strength, balance, spasticity, and mobility [21, 42, 44–48, 50]. Improvement in muscular strength attributed to WBVE was found in three studies [45, 50, 56]. van Nes et al. [43] reported comparable gains in muscle strength in 23 stroke patients and 23 elderly controls in which both groups received WBVE. However, most of the studies have not found such effects [21, 47, 48]. Among the positive results, Boo et al. (2016) [56] performed WBVE training in a sitting position (<30 Hz) and improved muscle tone in chronic stroke patients, although results should be considered with caution as the study included a limited number of subjects and there was no control group. Further, Tihanyi et al. [45] reported increased leg muscle strength after 4 weeks of conventional rehabilitation therapy in combination with WBVE. By contrast, Tankisheva et al. [50] used a frequency ranging from 35 to 45 Hz and found significant increments in the isometric knee extension strength after 6 weeks of WBVE training. In view of the used frequency it was suggested that intensive WBVE might potentially be a feasible way to increase muscle strength in adults with chronic stroke. In the same line, Liao et al. [61] suggested that higher WBVE intensities should be more effective than lower intensities in improving leg muscle strength. One possible explanation for these changes can be attributable to differences in muscle activation. Thus, recently Huang et al. [59] reported that WBVE at 30–40 Hz would be more appropriate to activate leg muscles. Similarly, various studies that assessed the effect of WBVE on muscle activation in stroke patients reported increments of the maximal voluntary contraction (10-25%) with frequencies between 20 and 30 Hz [53, 60]. The same group [57] recently performed a study on 30 stroke patients submitted to different WBVE conditions: (a) low-intensity WBVE (20 Hz frequency with 0.60 mm amplitude) and (b) high-intensity WBVE (30 Hz, 0.44 mm). Participants performed 8 dynamic exercises (3 sets of 45 each exercise) resulting in a significant increase in EMG amplitude of leg muscles, especially at higher WBVE intensities. Additionally, the muscle activity in the paretic leg achieved a greater relative activation compared with the nonparetic side. By contrast, Liao et al. [62] delivered WBVE training three times a week for a total of 30 sessions comparing low (20 Hz, 1 mm) or high (30 Hz, 1 mm) magnitude of vibration. Authors indicated that none of the protocols confer additional therapeutic effect for strength outcomes. A recent meta-analysis came to the same conclusion and showed that WBVE induced no significant effect on isometric and eccentric knee extension strength among individuals with stroke [62]. In another review [63] also assessed the effects of WBVE training on chronic stroke patients and concluded that WBVE training had no beneficial effects in muscle strength (isometric knee flexion or extension strength). One possible explanation of these inconsistencies might be the insufficient stimulus to induce a tonic vibration reflex and/or muscle spindles.

A decline of neuromotor performance is associated with reduced postural control, muscle power, and mobility, which is known to increase risk of falling [64]. However, a limited number of studies have assessed balance and falls in individuals with stroke [21, 42, 44, 46–50]. In 2004, van Nes et al. [43] published the first study which aimed to assess postural control in 23 chronic stroke patients. After 6 weeks of WBVE significant improvements in standing balance with the eyes closed and proprioception were observed [44]. Years later, Lau et al. (2012) [47] examined the efficacy of WBVE on neuromotor performance and risk of falling in chronic stroke patients. Forty-one participants received 9–15 min of WBVE (20–30 Hz, 0.44–0.60 mm) while performing dynamic leg exercises three times a week for 8 weeks. The study showed that WBVE was not effective in reducing the incidence of falls in patients with chronic stroke. Lee (2015) [52] also investigated the effects of WBVE on the motor function and balance. On this occasion vibration was delivered in the horizontal direction (15 min/day, 3 times/week, 6 weeks; 1-3 Hz, 30 mm) followed by conventional rehabilitation (30 min/day, 5 times/week, 6 weeks) leading to significant improvements in the scores of the Berg Balance Scale. Hwang et al. (2016) [54] assessed the immediate effect of WBVE on postural sway. In this study, when vibration was delivered at 10 Hz nonsignificant changes in postural sway were observed while with 40 Hz the postural sway in the mediolateral direction was improved. However, the study showed that WBVE was not helpful for improving the immediate balance ability of chronic stroke patients. The sample size in most of these studies was relatively small and relatively shorts (3-12 weeks of training). Only one of these studies reported positive results [63]. A recent systematic review revealed that the effects of WBVE on muscle strength and mobility performance remain inconclusive on patients with stroke [60]. Authors considered that these discrepancies can also be due to the differences in WBVE parameters (i.e., frequency, duration, and type of exercises) but also to the characteristics of participants across studies. Therefore, the evidence is insufficient to support the use of WBVE training in improving balance. In general, WBVE training yielded similar results on postural control compared with other types of physical activity [44, 46].

### 2.2. Effects of WBVE on the Mobility of Individuals Poststroke

Improvements in mobility have also been observed after WBVE in different studies [42, 46, 51]. Merkert et al. [46] reported better performance in the TUG test in the WBVE group after 3 weeks of training. Silva et al. (2014) [51] also reported positive effects of WBVE in gait performance (Six-Minute Walk Test and TUG test) in stroke patients. Acute significant improvements in walking speed were observed by Chan et al. [42] after a WBVE stimulus (12 Hz and amplitude of 4 mm for 20 minutes). Recently Choi et al. (2017) [58] investigated the effect of WBVE combined with treadmill training on walking performance in patients with chronic stroke. The study demonstrated that this training protocol improves the walking performance (i.e., walking speed, step length, or stride length). By contrast, no significant effects were found for the TUG [21], gait speed [21, 47], or Six-Minute Walk Test [21, 47] compared with a control therapy or sham WBVE stimulation.

### 2.3. Effects of WBVE on Spasticity Poststroke

Reductions in spasticity have been reported after WBVE [42, 49]. Resistance to passive movements in the ankle and knee on the paretic side was estimated according to the Modified Ashworth Scale [21, 42, 49, 50] and by the subjective experience of the influence of ankle spasticity on ambulation (using a visual analogue scale) [42]. After a single WBVE session (10 min, 12 Hz, and 4 mm), a significant decrease in ankle spasticity associated with improved mobility and speed has been observed [42]. Pang et al. [49] also reported improvements on knee spasticity. On the other hand, Brogärdh et al. (2012) [21] reported no significant treatment effect of WBVE on leg spasticity compared with sham WBVE. This finding was later supported in the findings of Tankisheva et al. (2014) [50].

### 2.4. Effects of WBVE on Cardiovascular Responses Poststroke

Only one study has explored the acute effect of different WBVE protocols on cardiovascular responses in the stroke population [53]. A modest significant increase of oxygen consumption and heart rate was found. Finally, a recent study [54] used 3 sessions/week of 5 min WBVE (4 weeks; 22-26 Hz, 2.1–6.5 mm) to assess the efficacy of short-term WBVE training on indices of arterial stiffness, although nonsignificant improvements were observed.

## 3. Summary

At this point we can conclude that there is no solid evidence confirming the beneficial effects of WBVE among people with stroke compared with either other types of physical activities or sham WBVE. Despite this, a modality of exercise could be an alternative for those clinicians who want to prescribe WBVE as part of rehabilitation in patients unable or unwilling to participate in traditional exercise training. In any case, further research should be performed in this area.

## 4. Future Research Directions

Possible explanations for the lack of significant differences between the groups include the discrepancies in WBVE protocols used.  Table 1 shows the different characteristics of the included studies in order to compare the variables used. It would be interesting to compare the effects of different WBVE frequencies, intensities, number of training sessions per week, and exercises used (e.g., dynamic versus static exercises) to assist the prescription of WBVE for people living with stroke. Further research is required to compare different WBVE parameters. Perhaps a more homogeneous group of patients with more severe neuromotor impairment would benefit more from the WBVE training [47]; thus, future directions also included the necessity to test the feasibility and efficacy of using WBVE in a more homogeneous sample of stroke patients.

## Figures and Tables

**Table 1:** Whole body vibration exercises characteristics of the selected studies.

**Study**	**N**	**Age (years)**	**Frequency (Hz)**	**PPD (mm)**	**Type vibration and device**	**Position**	**Duration (weeks)**	**Number of sessions**	**Number of bouts**	**Time series**	**Rest between series**
van Nes (2004) [43]	23	58.1 ± 11.4	30	3	Galileo (Side-Alternating)	Squat (knees and hips slightly bent)	-	Single	4	45 s	60 s

van Nes (2006) [44]	53	61.1 ± 10.1	30	3	Galileo (Side-Alternating)	Squat (“slight” flexion hips and knees)	6	30	4	45 s	60 s

Tihanyi et al. (2010) [45]	20	58.6 ± 6.3	20	2.5	Synchronous Vertical	Squat (Knee flexed 80°)	4	12	6	60 s	60 s

Merkert et al. (2011) [46]	66	74.5 ± 8.5	20-45	-	Vibrosphere	Sitting (Knees and hip flexed 90°)	3	15	2	90 s	15-90 s

Brogärdh et al. (2012) [21]	31	62.6 ± 7.3	25	3.75	Synchronous Vertical	Squat (Knee flexed 45-60°)	6	12	4-12	40-60 s	60 s

Chan et al. (2012) [42]	30	56.1 ± 11.0	12	4	Synchronous Vertical	Squat (Joints angles not reported)	-	Single	2	10 min	60 s

Lau et al. (2012) [47]	82	57.3 ± 11.3	20-30	0.44-0.60	Jet-Vibe System (synchronous)	Different exercises	8	24	6	90-150 s	3-4.5 min

Tankisheva et al. (2013) [10]	15	61.6 ± 9.2	35-45	1.7-2.5	Powerplate (Synchronous)	Different exercises	6	18	5-17	30-60 s	-

Marín et al. (2013) [48]	20	63.2 ± 9.4	5-21	2-3	Side-alternating Vertical	Squat (Knee flexed 30°)	12	17	4-7	30-60 s	60 s

Pang et al. (2013) [49]	82	57.3 ± 11.3	20-30	0.44-0.60	Jet-Vibe System (synchronous)	Different exercises	8	24	6	90-150 s	3-4.5 min

Tankisheva et al. (2014) [50]	15	57.4 ± 13.0	35-40	1.7-2.5	Powerplate (Synchronous)	Different exercises	6	18	5-17	30-60 s	-

Silva et al. (2014) [51]	38	60.7 ± 11.8	50	2	Extream 1000	Squat (Knee flexed 30°)	-	Single	4	60 s	60 s

Lee (2015) [52]	26	59.3 ± 13.2	1-3	30	(Side-alternating Horizontal)	Squat (knees and hips slightly bent)	6	18	1	10 min	-

Liao et al. (2015) [53]	36	57.3 ± 10.7	20-30	0.44-0.60	Jet-Vibe System (synchronous)	Different exercises	-	Single	3	8 x 10 s	60 s

Hwang et al. (2016) [54]	14	68.2 ± 1.3	10-40	-	Vibro wedge (Synchronous)	Squat (Joints angles not reported)	-	Single	1	10 min	-

Yule et al. (2016) [55]	6	50.5 ± 14.5	22-26	2.1-6.5	Galileo (Side-Alternating)	Squat (Knee flexed 70°)	4	12	5-7	60 s	60 s

Boo et al. (2016) [56]	14	50.4 ± 12.4	<30	-	Vibro wedge (Synchronous)	Sitting (Knees and hip flexed 90°)	8	45	1	10 min	-

Liao et al. (2017) [57]	30	56.8 ± 10.1	20-30	0.44-0.60	Jet-Vibe System (synchronous)	Different exercises	-	Single	15	45 s	60 s

Choi et al. (2017) [58]	30	51.9 ± 8.3	20-30	3	Galileo (Side-Alternating)	Different exercises	6	18	6	45 s	60 s

Huang et al. (2018) [59]	34	62.3 ± 6.7	20-40	0.8-1.5	Fitvibe Excel (synchronous)	Different exercises	-	Single	18	20 s	60 s

N- size of the experimental group, PPD- peak to peak displacement
